# Decoding Bromodomain and Extra-Terminal Domain Protein-Mediated Epigenetic Mechanisms in Human Uterine Fibroids

**DOI:** 10.3390/ijms262412144

**Published:** 2025-12-17

**Authors:** Qiwei Yang, Somayeh Vafaei, Ali Falahati, Azad Khosh, Mervat M. Omran, Tao Bai, Maria Victoria Bariani, Mohamed Ali, Thomas G. Boyer, Ayman Al-Hendy

**Affiliations:** 1 Department of Obstetrics and Gynecology, University of Chicago, Chicago, IL 60637, USA; somayeh.vafaei@gmail.com (S.V.); mervatomran@bsd.uchicago.edu (M.M.O.); bariani@northwestern.edu (M.V.B.); mohamed.ali@bsd.uchicago.edu (M.A.); aalhendy@bsd.uchicago.edu (A.A.-H.); 2 Poundbury Cancer Institute for Personalised Medicine, Dorchester DT1 3BJ, UK; ali.falahati@nhs.net; 3 Department of Molecular Medicine, Institute of Biotechnology, University of Texas Health Science Center at San Antonio, San Antonio, TX 78229, USA; khosh@livemail.uthscsa.edu (A.K.); boyer@uthscsa.edu (T.G.B.); 4 Obstetrics and Gynecology, Feinberg School of Medicine, Northwestern University, Chicago, IL 60611, USA; tao.bai@northwestern.edu; 5 Department of Medical Sciences, Khalifa University, Abu Dhabi P.O. Box 127788, United Arab Emirates

**Keywords:** uterine leiomyoma, bromodomain and extra-terminal domain, bromodomain-containing protein, BET protein inhibitor, cell viability, cell cycle arrest, transcriptome, ECM, inflammation response, epigenome

## Abstract

Uterine Fibroids (UFs) are the most common benign tumors in women of reproductive age, affecting ~77% of women overall and are clinically manifest in ~25% by age 50. Bromodomain and extra-terminal domain (BET) proteins play key roles in epigenetic transcriptional regulation, influencing many biological processes, such as proliferation, differentiation, and DNA damage response. Although BET dysregulation contributes to various diseases, their specific role in the pathogenesis of UFs remains largely unexplored. The present study aimed to determine the expression pattern of BET proteins in UFs and matched myometrium and further assess the impact of BET inhibitors on UF phenotype and epigenetic changes. Our studies demonstrated that the levels of Bromodomain-containing protein (BRD)2 and detection rate of BRD4 were significantly altered in UFs compared to matched myometrium, suggesting that aberrant BET protein expression may contribute to the pathogenesis of UFs. To investigate the biological effects of BET proteins, two small-molecule inhibitors, JQ1 and I-BET762, were used to assess their impact on UF cell behavior and transcriptomic profiles. Targeted inhibition of BET proteins markedly reduced UF cell viability compared with myometrial cells and induced cell cycle arrest. Unbiased transcriptomic profiling coupled with bioinformatic analysis revealed that BET inhibition altered multiple biological pathways, including G2M checkpoint, E2F targets, mitotic spindle, mTORC1 signaling, TNF-α signaling via NF-κB, and inflammatory response, as well as reprogrammed the UF cell epigenome. Notably, BET inhibition decreased the expression of several genes encoding extracellular matrix (ECM) proteins, a hallmark of UFs. Collectively, these results support that BET proteins play a pivotal role in regulating key signaling pathways and cellular processes in UFs. Targeting BET proteins may therefore represent a promising non-hormonal therapeutic strategy for UF treatment.

## 1. Introduction

Uterine Fibroids (UFs), also known as leiomyomas, are the most common benign smooth muscle tumors of the uterus and represent a significant gynecological and public health concern among women of reproductive age. Epidemiological studies estimate that up to 77% of women will develop UFs during their lifetime, with approximately one in four experiencing clinically significant symptoms by the age of 50 [1,2,3]. These tumors are often multifocal and display remarkable heterogeneity in their size, number, and cellular composition, even within a single uterus. Such diversity contributes to a wide spectrum of clinical manifestations, including abnormal uterine bleeding, pelvic pain, infertility, recurrent pregnancy loss, and adverse obstetric outcomes such as preterm birth. UFs are the leading indication for hysterectomy in the United States [4], contributing substantially to health care costs and diminished quality of life worldwide [5]. Despite their high prevalence and socioeconomic burden, the molecular mechanisms that drive UF initiation, growth, and recurrence remain incompletely understood.

Emerging evidence suggests that UFs are hormone-dependent tumors primarily regulated by estrogen and progesterone. However, hormonal therapy provides only temporary symptom relief and is often associated with adverse effects, highlighting the urgent need to elucidate non-hormonal molecular mechanisms underlying UF development. In recent years, attention has shifted toward the role of epigenetic dysregulation, reversible modifications that influence gene expression without altering the DNA sequence. Epigenetic mechanisms, including DNA methylation, histone modification, and non-coding RNA-mediated regulation, have been increasingly recognized as pivotal contributors to the aberrant gene expression profiles characteristic of UFs [6,7]. Among these mechanisms, the function of epigenetic “reader” proteins has gained particular interest for their ability to interpret chromatin modifications and translate them into specific transcriptional programs.

The bromodomain and extra-terminal domain (BET) family of proteins, comprising bromodomain-containing protein 2 (BRD2), BRD3, BRD4, and bromodomain testis-specific protein (BRDT), represents a prominent class of chromatin readers that bind acetylated lysine residues on histone tails. By recognizing these acetylation marks, BET proteins recruit transcriptional complexes and facilitate activation of genes involved in critical biological processes such as cell cycle regulation, proliferation, differentiation, apoptosis, and the DNA damage response [8,9,10,11]. Dysregulation of BET protein expression or activity has been implicated in multiple pathological conditions, including tumorigenesis, inflammation, cardiovascular disease, and tissue fibrosis [12,13]. Histone acetylation and deacetylation operate within a dynamic equilibrium regulated by acetyltransferases and deacetylases, providing binding signals for “reader” proteins and altering cellular processes. Pharmacological inhibition of BET proteins using small-molecule inhibitors, such as JQ1 and I-BET762, has demonstrated promising therapeutic potential in preclinical models of cancer and fibrotic disorders [14,15,16].

Despite this growing evidence, the role and regulatory mechanism of BET proteins in the pathogenesis of UFs remains poorly understood, particularly regarding their contribution to the epigenetic and transcriptional reprogramming of UF cells. Given the critical functions of BET proteins in transcriptional control and cellular homeostasis, we hypothesized that aberrant expression or activation of BET family members contributes to UF development and maintenance by modulating key signaling and epigenetic pathways. To test this, we examined BET protein expression in UFs and matched myometrial tissues and assessed the molecular and biological effects of BET inhibition using selective small-molecule inhibitors JQ1 and I-BET762. Our findings demonstrate that BRD2 and BRD4 are significantly changed in UFs and that BET inhibition reduces UF cell viability, induces cell cycle arrest, and alters global transcriptomic and epigenetic profiles. Notably, BET inhibition suppresses the expression of extracellular matrix (ECM)-related genes, a hallmark of fibroid pathology.

## 2. Results

### 2.1. BET Proteins Are Aberrantly Upregulated in Uterine Fibroids

**Figure 1:**
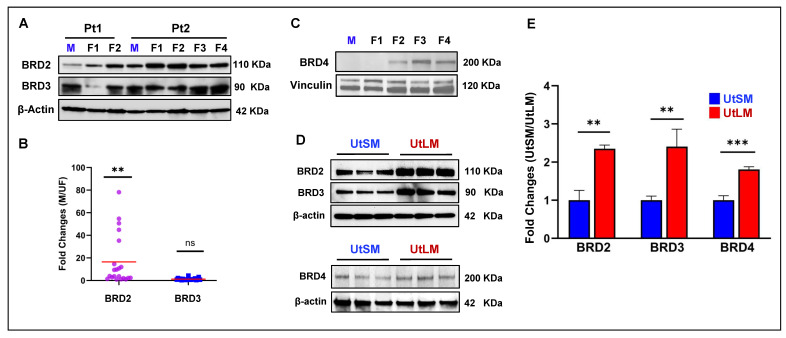
Comparison of BET protein levels between uterine fibroid and myometrium tissues (M) and cells. (**A**) Immunoblot analysis was performed to determine the levels of BET proteins in UFs (n = 21) and myometrium tissues (n = 7). The protein levels of BRD2 were significantly upregulated in UFs compared to matched myometrium tissues. (**B**) The BET protein levels of BRD2 and BRD3 were quantified from Figure 1A and supplemental Figure S1A using Image J software (version 1.52r) and presented as fold changes. (**C**) A representative image of BRD4 protein expression from one patient in matched M and UF tissues. A total of 6 M and 17 UF tissues samples were analyzed. (**D**) The protein levels of BRD2–4 in M and UF cells. (**E**) Comparison of BRD2–4 gene expression between M and UF cells using real-time PCR. Relative mRNA expression levels of BRD family genes were measured in UtSM and UtLM cells using quantitative real-time PCR. Gene expression was normalized to the housekeeping gene 18S, and data are presented as mean ± standard error of the mean (SEM) from at least three independent experiments. Statistical significance between UtSM and UtLM cells was determined using Student’s *t*-test. The results indicate differential expression of specific BRD family genes between UtSM and UtLM cells. ** *p* < 0.01; *** *p* < 0.001; ns: no significant difference, Pt: patient, M: myometrium tissue, F: fibroids, UtLM: immortalized UF cell line, UtSM: immortalized myometrial cell line.

We first measured the levels of crucial BET proteins, including BRD2 and BRD3, in human UFs (n = 21 from 7 UF patients) and matched myometrial tissues (n = 7). As shown in Figure 1A,B, and Supplemental Figure S1A, among the 21 UFs analyzed, 82% (18/22, *p* < 0.01) exhibited upregulation of BRD2 compared to matched myometrium. In contrast to BRD2, the protein levels of BRD3 exhibited no significant difference between UFs and matched myometrium tissues (Figure 1A,B). For BRD 4 (Figure 1C, Supplemental Figure S1B), a total of 6 M and 17 UF tissues were analyzed. Since some control samples had signals below the limitation of detection, we used Fisher’s exact test to compare the detection rate of BRD4. The analysis revealed a significantly higher detection rate of BRD4 in UFs compared with myometrial tissues (66.7%, UF: 23.5%, *p* < 0.05).

Next, we compared the protein levels of BET proteins between M and UF cells. As shown in Figure 1D, UF cells (UtLM) showed higher protein levels of BRD2, BRD3, and BRD4 compared to uterine myometrial cells (UtSM). qPCR analysis revealed differential expression of BET family members in human UF UtLM cells compared with UtSM cells. The mRNA levels of BRD2, BRD3, and BRD4 were significantly upregulated in UtLM cells relative to UtSM cells (Figure 1E), suggesting that increased BET protein expression may result from transcriptional activation in UF cells. These findings support the hypothesis that dysregulation of BET proteins contributes to the molecular pathogenesis of UFs.

### 2.2. Inhibition of BET Proteins Altered UF Cell Proliferation and Induced Cell Cycle Arrest

**Figure 2:**
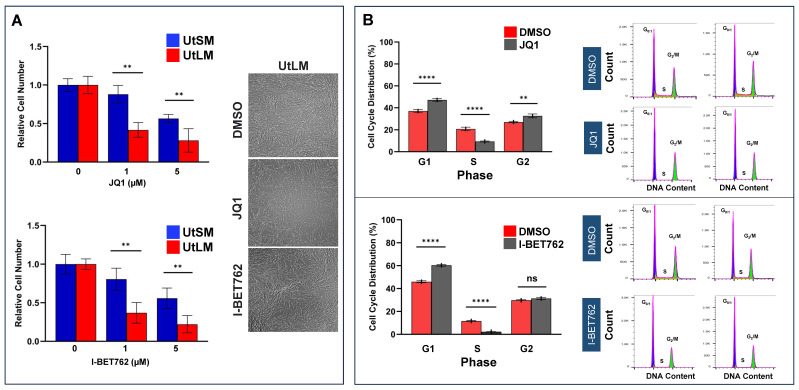
Targeted inhibition of BET proteins potently decreases cell growth and induces cell cycle arrest in UtLM. (**A**) Cell viability assay was performed in UtLM cells in presence or absence of JQ1 and I-BET 762 for 48 h, respectively (left panel). Trypan blue exclusion assay was used to quantify viable cell numbers. The portion of trypan blue—positive (dead) cells after 48 h of treatment with 5 µM inhibitors is less than 7%, indicating minimal cell death. Moreover, JQ1 and I-BET 762 treatments decreased cell growth in a concentration-dependent manner The right panel showed the morphology of UtLM cells in the presence or absence of 5 µM JQ1 or I-BET762 for 48 h. UtLM: UF cell line; UtSM: myometrial cells, ** *p* < 0.01. (**B**) Targeted Inhibition of BET proteins induced cell cycle arrest in UF UtLM cells. UtLM cells were cultured in the absence or presence of BET protein inhibitors (5 µM JQ1, I-BET762) for 48 h. The flow cytometry was used to determine the percentages of cell population in the different phases of the UF cell cycle in response to BETi treatments. G1 and G2 phase histogram peaks were separated by the S-phase distribution in UF cells. ** *p* < 0.01, **** *p* < 0.001 compared between treated groups and control. ns: no significant difference. The values represent the relative number of viable (unstained) cells in each treatment group compared with vehicle-treated cells, and do not indicate percent viability.

The small molecule inhibitors JQ1 and I-BET762 have been shown to potently exhibit anti-proliferative effects in various types of cancer by binding competitively to bromodomains [17,18,19]. In this study, we first examined the biological impact of BET protein inhibition on UF cell viability. Trypan blue exclusion assay was used to quantify viable cell numbers. The values represent the relative number of viable (unstained) cells in each treatment group compared with vehicle-treated cells. According to the assay, BETis do not induce a cytotoxic effect. The portion of trypan blue—positive (dead) cells after 48 h of treatment with 5 µM inhibitors is less than 7%, indicating minimal cell death. As shown in Figure 2A, JQ1 and I-BET762 treatments decreased cell growth in a concentration-dependent manner, demonstrating an antiproliferative rather than cytotoxic effect in both UF and M cell lines (Figure 2A). Moreover, targeted inhibition of BET proteins showed stronger inhibitory effects on UF cell growth compared to M cells at both 1 and 5 µM BETis. On the right panel of Figure 2A, the morphology of UF cells in the presence or absence of BET protein inhibitors is shown.

To validate the anti-proliferative effect of BET protein inhibitors on UF cell growth, we measured cell cycle changes with or without JQ1/I-BET762 treatments. As shown in Figure 2B, JQ1 and I-BET762 treatments caused an increased accumulation of cells at the G1 phase and a corresponding decrease in the S phase, indicating blockage of cell cycle progression. The percentage of cells in the G0/G1 phase increased from 37% to 47.2% and from 46% to 60.4% in response to 5 µM JQ1 and I-BET762 treatment, respectively. Accordingly, treatment with JQ1 and I-BET762 also led to a a marked reduction in the S-phase population compared with the vehicle control (9.4% vs. 20.8%; 2.3% vs. 11.6%, respectively). These results are consistent with our observation that JQ1 and I-BET762 suppressed UF cell proliferation, and the inhibition of UtLM cell proliferation by BET protein inhibitors is at least partly due to cell cycle arrest.

### 2.3. BET Proteins Inhibition Causes Extensive Changes in the UF Cell Transcriptome

**Figure 3:**
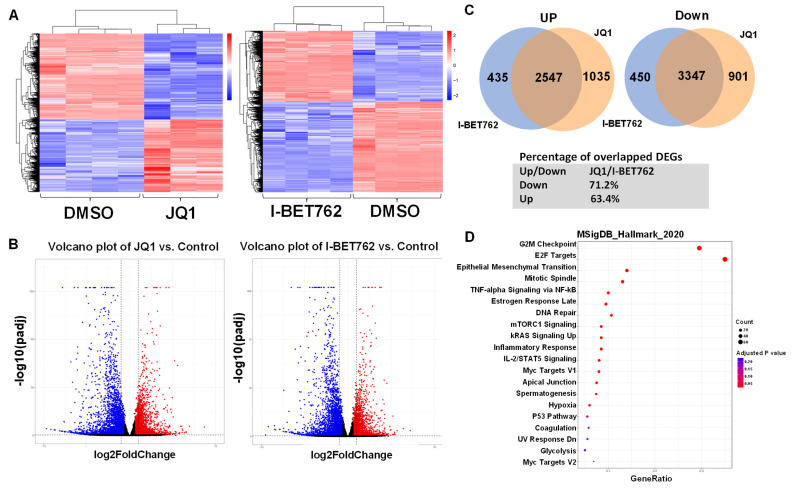
Targeted inhibition of BET proteins elicits a distinct gene expression pattern in UF cells. (**A**) Heat maps are presented to cluster DEGs between JQ1 vs. vehicle group and I-BET762 vs. vehicle group. (**B**) Volcano plot revealing the distribution of the DEGs between JQ1 and I-BET762 vs. control. Each dot represents an individual gene. Red dots indicate significantly upregulated genes, and blue dots indicate significantly downregulated genes following BETi treatment, based on the defined statistical thresholds. The cutoff value is 1.5-fold with an FDR < 0.05. (**C**) Overlapped DEGs between JQ1 and I-BET762 treatment groups. (**D**) The gene set enrichment analysis was performed using the molecular signatures database (MSigDB).

To investigate the mechanistic basis for the inhibitory action of BET protein inhibitors (BETis), JQ1 and I-BET762, in UF cells, RNA-sequencing analysis was performed in control (n = 4), JQ1 (n = 3), and I-BET762 (n = 4) treated UF UtLM cells. JQ1 treatment yielded 7863 differentially expressed genes (DEGs) (4267 down, 3595 up). I-BET762 treatment generated 6814 DEGs (3815 down, 2999 up). For JQ1 treatment, 12.4% and 14.7% of genes were significantly up or down-regulated, respectively. For I-BET762 treatment, 10.7% and 13.7% of genes exhibited significant up or down-regulation, respectively. Heatmap analysis further demonstrated a distinct expression pattern in UtLM cells treated with JQ1 (Figure 3A, left panel) and I-BET762 (Figure 3A, right panel) compared to the control group. Volcano plot analysis revealed the distribution of changes in response to JQ1 and I-BET762 treatment (Figure 3B, left and right panels).

Next, we determined the number of overlapping DEGs within the JQ1 and I-BET762 treatment groups and found that 63.4% and 71.2% DEGs were commonly regulated (up and down) between the two groups (Figure 3C). To gain further insight into the biological changes elicited by BET protein inhibition, we performed Hallmark gene set enrichment analysis and identified several pathways that were significantly and commonly altered by JQ1 and I-BET762 treatment, including G2M checkpoint, E2F targets, mitotic spindle, TNF-alpha signaling via NF-kB, mTORC1 signaling, inflammatory response, among others (Figure 3D). This analysis suggested that JQ1 and I-BET762 inhibitors suppress UF cell growth through multiple cellular mechanisms.

### 2.4. Inhibition of BET Proteins Altered Gene Expression in Multiple Biological Processes

**Figure 4:**
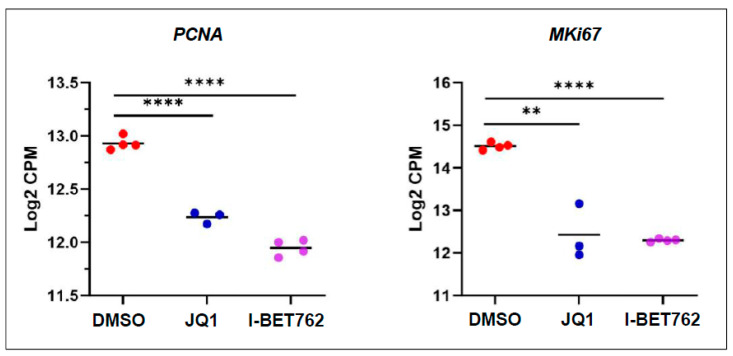
Comparison of *PCNA* and *MKi67* mRNA expression between BETi- and vehicle-treated UF cells. The expression of *PCNA* and *MKi67*, cell proliferation markers, were significantly decreased in BETi-treated UF cells. ** *p* < 0.01, **** *p* < 0.0001. CPM: Counts Per Million; *MKi67*: the gene that encodes the Ki67 protein.

To determine the correlation between the expression of key genes and biological effects, we first examined the expression of genes related to cell proliferation markers. As shown in Figure 4, the RNA expression of *PCNA* and *MKi67* was significantly downregulated in BETi-treated UtLM cells compared to control cells (vehicle-treated UtLM cells).

**Figure 5:**
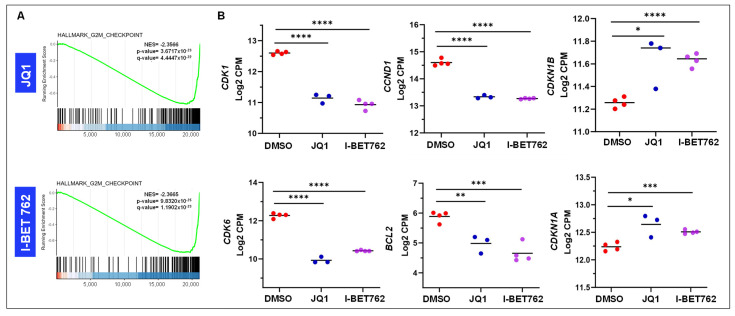
BET inhibitors alter the expression of cell cycle-related genes in UF cells. RNA-seq analysis revealed that treatment with BET inhibitors (BETis) modulated the expression of key cell-cycle-regulatory genes in UF cells. (**A**) The enrichment plot shows the distribution of genes from the HALLMARK_G2M CHECKPOINT gene set across the ranked gene list. The green curve represents the running enrichment score (ES), indicating coordinated downregulation of G2M checkpoint genes upon BETi treatment. The normalized enrichment score (NES) and statistical significance values are shown on the plot. (**B**) BETis upregulated the expression of *CDKN1A* and *CDKN1B*, while downregulating *CDK1*, *CDK6*, *CCND1*, and *BCL2*. * *p* < 0.05; ** *p* < 0.01; *** *p* < 0.001; **** *p* < 0.0001.

Next, we identified gene sets impacting biological processes. We first checked the expression of cell cycle-related genes (Figure 5A). As shown in Figure 5B, the magnitude of expression of cell cycle-related genes, including *CDK1*, *CCND1*, *CDK6*, and *BCL2*, was significantly downregulated in BETi-treated UtLM cells compared to control cells. In contrast, the BETis increased the expression of *CDKN1A* and *CDKN1B* in UtLM cells. These results are consistent with our observation that BETis suppressed the UF cell viability and induced cell cycle arrest in UtLM cells. To validate the DEGs associated with cell cycle progression, we selected several key genes and performed RT-PCR analysis to confirm our findings. Our RT-qPCR data showed that the expression of *PCNA*, *CCND1*, and *CDK1* was significantly decreased in UtLM cells treated with JQ1 and I-BET762 compared to vehicle control (Supplemental Figure S2A). These results suggest that BET inhibition induces cell cycle arrest and reduces pro-survival signaling in UF cells.

**Figure 6:**
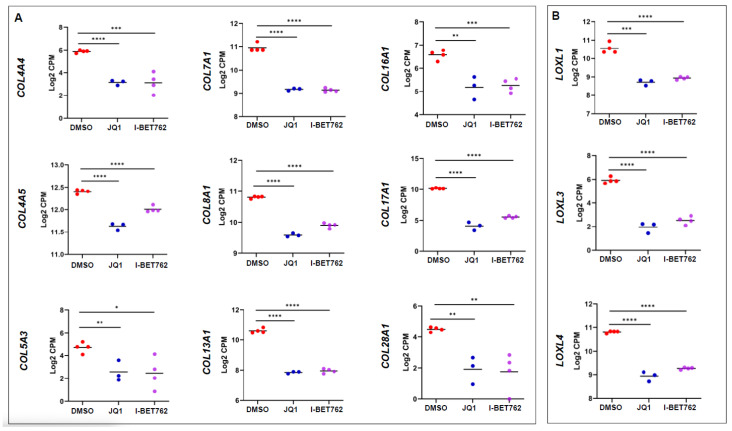
BETis altered the expression of ECM and ECM-related genes in UF cells. (**A**) RNA-seq analysis revealed that BETi treatments significantly downregulated the expression of *COL4A4*, *COL4A5*, *COL5A3*, *COL7A1*, *COL8A1*, *COL13A1*, *COL16A1*, *COL17A1*, and *COL28A1* in UF cells. (**B**) BETis downregulated the expression of LOXL family, including *LOXL1*, *LOXL3*, and *LOXL4* in UF cells * *p* < 0.05; ** *p* < 0.01; *** *p* < 0.001; **** *p* < 0.0001.

Excessive ECM accumulation and abnormal ECM remodeling are critical for UFs [20,21,22]. Excessive ECM depositions can contribute to mechanotransduction, therefore regulating downstream signaling and leading to the pathogenesis of UFs. In this study, we demonstrated that the expression of several ECM genes, including *COL4A4*, *COL4A5*, *COL5A3*, *COL7A1*, *COL8A1*, *COL13A1*, *COL16A1*, *COL17A1*, and *COL28A1*, were significantly downregulated, suggesting that BETis may reverse the UF phenotype via inhibiting ECM production (Figure 6A). Intramolecular and intermolecular Lysyl oxidase cross-links can form mature collagen fibrils that impact functional signals in UFs [23,24]. Notably, our data revealed that targeted inhibition of BET proteins reduced the RNA expression of multiple LOXL family members, including *LOXL1*, *LOXL3*, and *LOXL4* (Figure 6B), suggesting that BET proteins may be involved in the mature formation of collagen fibrils.

**Figure 7:**
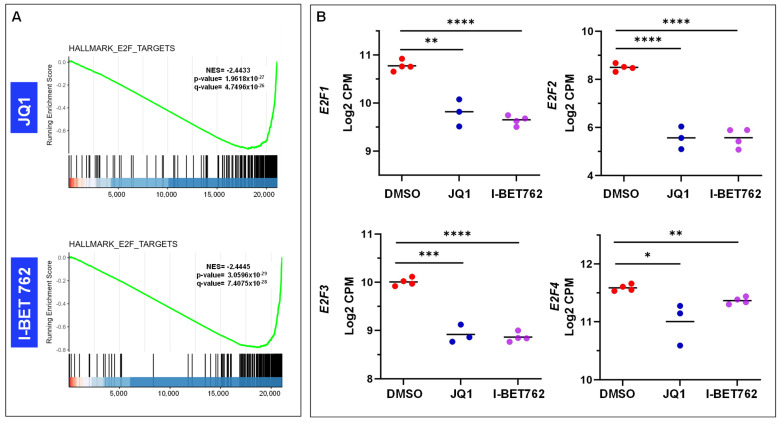
BETis altered the expression of E2F family genes in UF cells. (**A**) The enrichment plot shows the distribution of genes from the HALLMARK E2F TARGETS gene set across the ranked gene list. The green curve represents the running enrichment score, indicating coordinated downregulation of E2F TARGETS` genes upon BETi treatment. The normalized enrichment score (NES) and statistical significance values are shown on the plot. (**B**) RNA-seq analysis revealed that BETi treatment significantly downregulated the expression of *E2F1*, *E2F2*, *E2F3*, and *E2F4* in UF cells. * *p* < 0.05; ** *p* < 0.01; *** *p* < 0.001; **** *p* < 0.0001.

Since the pathway of E2F targets was enriched in UtLM cells treated with JQ1/I-BET762 (Figure 7A), we determined the expression of 4 key E2F genes, *E2F1*, *E2F2*, *E2F3*, and *E2F4*. As shown in Figure 7B, the expression of *E2F1–4* exhibited a significant decrease in UtLM cells treated with JQ1/I-BET762 compared to the control group (DMSO).

**Figure 8:**
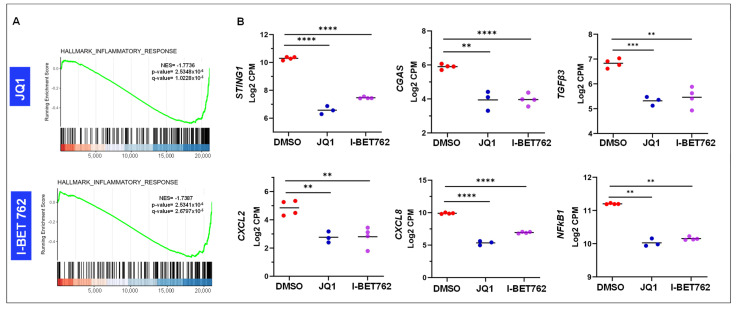
BETis altered the expression of inflammatory response genes in UF cells. (**A**) The enrichment plot shows the distribution of genes from the HALLMARK_INFLAMMATORY_RESPONSE gene set across the ranked gene list. The green curve represents the running enrichment score, indicating coordinated downregulation of inflammatory response–associated genes upon BETi treatment. The normalized enrichment score (NES) and statistical significance values are shown on the plot. (**B**) RNA-seq analysis revealed that BETi treatment significantly downregulated the expression of several key inflammation-related genes, including *NF-κB1*, *TGFβ3*, *CXCL2*, *CXCL8*, *STING1*, and *CGAS* in UF cells. ** *p* < 0.01; *** *p* < 0.001; **** *p* < 0.0001.

We noted that inflammatory response pathway was enriched in BETi-treated UF cells (Figure 8A). Accordingly, the RNA expression of key genes, including *TGFβ3*, *NF-kB1*, *CXCL2*, *CXCL8*, *CGAS*, and *STING1*, was significantly reduced in BETi-treated UF cells compared to control group (DMSO) (Figure 8B). To validate the DEGs related to the inflammation merkers, we performed RT-qPCR and revealed that JQ1 and I-BET762 significanly decreased the expression of *NF-kB* and *STING1*, compred to vehicle control (Supplemental Figure S2B).

### 2.5. Inhibition of BET Proteins Altered Gene Expression Associated with Epigenetic Marks

**Figure 9:**
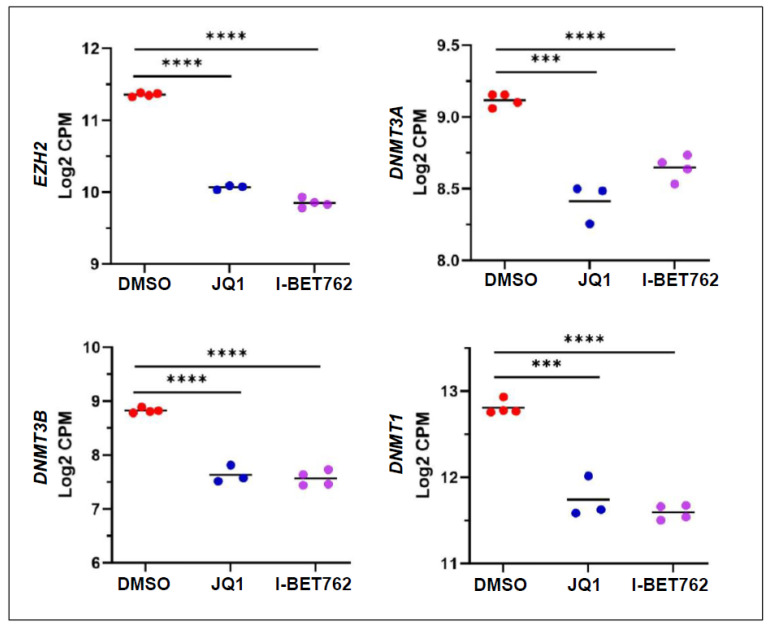
BETis altered the expression of genes encoding histone and DNA methylation regulators in UF cells. RNA-seq analysis revealed that BETi treatments significantly downregulated the expression of *EZH2*, *DNMT1*, *DNMT3A*, and *DNMT3B* in UF cells. *** *p* < 0.001; **** *p* < 0.0001.

To investigate if BETis reprogrammed the UF cell epigenome by modulating the expression of epigenetic regulators, we performed targeted gene analysis using our RNA-seq data and found that JQ1 and I-BET762 altered the RNA expression of several epigenetic genes. As shown in Figure 9, JQ1/I-BET762 reduced the expression of *DNMT3A*, *DNMT3B*, *DNMT1*, and *EZH2*, related to DNA and histone methylation.

**Figure 10:**
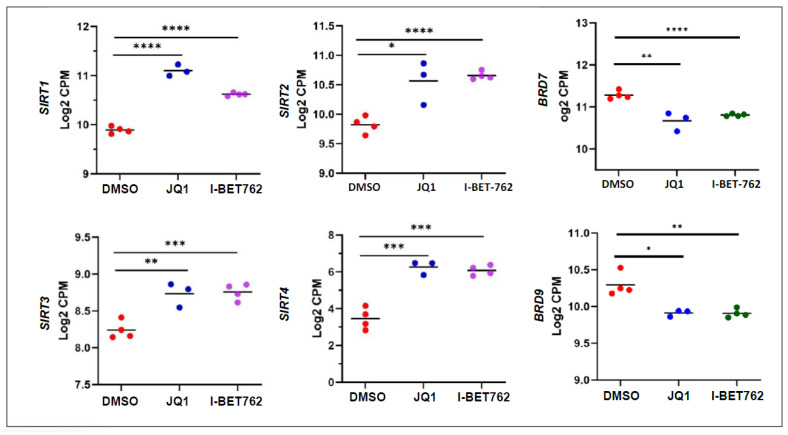
BETis altered the expression of genes encoding histone acetylation-related regulators in UF cells. RNA-seq analysis revealed that BETi treatment significantly downregulated the expression of *BRD7* and *BRD9*, and increased the expression of *SIRT1*, *SIRT2*, *SIRT3*, and *SIRT4* in UF cells. * *p* < 0.05; ** *p* < 0.01; *** *p* < 0.001; **** *p* < 0.0001.

We also observed that JQ1 and I-BET762 altered the expression of histone acetylation-related genes, including *SIRT1*, *SIRT2*, *SIRT3*, *SIRT4*, *BRD7*, and *BRD9* (Figure 10). The latter was recently identified to be involved in UF pathogenesis [25,26]. To validate the DEGs related to the epigenetic regulators, we selected three genes and performed RT-qPCR and revealed that JQ1 and I-BET762 significanly decreased the expression of *DNMT1* and *DNMT3A*, and increased the expression of *SIRT1*, compred to vehicle control (Supplemental Figure S2C).

### 2.6. Inhibition of BET Proteins Altered the Gene Expression Correlating to Histone Modifications

To investigate whether JQ1 and I-BET762 treatment led to transcriptional changes via epigenomic effects in UF cells, we performed enrichment analysis of epigenetic histone markers using the Enrichr web server (https://maayanlab.cloud/Enrichr/, accessed on 21 May 2023). As shown in Supplemental Figure S3, DEGs (up and down) between control and BETi-treated UtLM cells were correlated with histone modifications. Notably, genes commonly induced by JQ1/I-BET762 treatments were significantly linked to H3K4me3, H3K4me2, H3K79me2, H3K4me1, H3K29ac, and H3K27ac (Supplemental Figure S3A). Conversley, genes commonly suppressed by JQ1/I-BET762 treatment were preferentially associated with H3K27me3, and H3K4me1 (Supplemental Figure S3B). These analyses suggest that JQ1/I-BET762 treatments may alter the transcriptome via epigenetic mechanisms.

## 3. Discussion

UFs are a common and clinically significant condition that still lacks effective therapeutic options. This study provides novel insights into the role of BET proteins in UF pathogenesis and suggests a potential new therapeutic strategy for this poorly treated disease. Epigenetic alterations regulate gene expression beyond DNA sequence and are implicated in many diseases, including cancer [27]. Understanding how epigenetic regulators contribute to tumorigenesis is crucial for developing chromatin-targeted therapies [28,29,30,31]. Our findings reveal that the BET family protein BRD2 and BRD4, the key readers of lysine acetylation that regulates chromatin remodeling, is upregulated in UFs. Targeted inhibition of BET proteins induced cell cycle arrest, altered multiple biological pathways, and reprogrammed the pathological epigenome in UF cells.

Abnormal cell proliferation, combined with the excessive production of extracellular matrix deposition drives UF expansion. Epigenetic-targeted therapies have been shown potent anticancer effects [32,33,34]. The development and use of small chemical inhibitors are fundamental and critical to the preclinical evaluation of BET proteins as targets. Recently, several BET inhibitors have been developed with suppressive effects on many types of cancers [18,35,36,37,38,39]. In this study, we determined the effect of JQ1 and I-BET762, the specific BET protein inhibitors, on UF cells and demonstrated that JQ1/I-BET 762 treatment significantly inhibited UF cell proliferation, concomitant with increased cell cycle arrest. This is consistent with the previous observation that JQ1 suppresses tumor growth associated with a cell cycle arrest in ovarian cancer [40]. We identified several cell cycle-related components involved in the cell cycle progression in the context of inhibition of UF cell proliferation. Previously, several studies demonstrated that targeted inhibition of multiple crucial components, including BRD9 [19,36], suppressed UF cell proliferation via cell cycle arrest. In addition, several gene sets related to cell proliferation, such as G1/S transition, G2M checkpoint, and DNA replication, have been changed in response to the treatment of BRD9 inhibitors [25,26].

BET inhibition also reduced the expression of multiple ECM-related genes, indicating potential attenuation of UF fibrosis. Notably, BET inhibitors significantly decreased LOXL family expression, which mediates collagen cross-linking and matrix stiffening. These findings underscore the role of BET proteins in ECM remodeling and fibrosis in UFs.

E2F family members play a crucical role during the G1/S transition in mammalian cells. Several categories of proteins, including cyclins and CDKs are E2F transcriptional targets. In this study, we observed that the expression of *CDK1* and *CDK6* were significantly downregulated, which were correlated with decreased gene expression of transcription activators *E2F1*, *E2F2*, and *E2F3*. Notably, the expression of *E2F1–3* was significantly downregulated in the UtLM cells treated with BETis, suggesting that BETis impact the gene set of E2F targets.

Inflammation is another key contributor to UF pathogenesis [41,42]. Proinflammatory cytokines, including TGF-β, are upregulated in UFs and promote both inflammation and ECM deposition [43,44]. It is reported that BRD2 is essential for proinflammatory cytokine production in macrophages [45]. BRD2 and BRD4 physically associate with the promoters of inflammatory cytokine genes in macrophages. JQ1 can block this association and reduce IL-6 and TNF-a levels. These studies suggested that targeting the BET proteins could benefit hyperinflammatory conditions associated with high levels of cytokine production [45]. We observed that BETis treatments significantly decreased the expression levels of *TGF-β3*, which may attenuate cell proliferation, regulate ECM remodeling, and exert an anti-inflammation effect in UFs. NF-κB is a transcription molecule activated by various intra- and extra-cellular stimuli such as cytokines. NF-κB translocates into the nucleus and stimulates the expression of genes involved in a wide variety of cellular processes and functions. In UFs, the proinflammatory cytokine TNF-α activates the NF-κB pathways in UF cells. It is reported that BET proteins interact with NF-κB, which can co-regulate target genes [45]. Given the interaction between BET proteins and NF-κB, this suggests that BET inhibitors may suppress the inflammatory microenvironment in UFs through NF-κB pathway inhibition. Together, these results support the idea that BET inhibitors exert dual effects—controlling proliferation and mitigating chronic inflammation—making them attractive candidates for treating fibrotic and inflammatory diseases beyond UFs.

Previous studies reported that different epigenetic mechanisms coordinately regulate gene expression and function [46,47,48,49]. To explore the broader epigenetic impact of BET inhibition, we examined histone mark enrichment associated with DEGs after JQ1/I-BET762 treatment. DEGs were significantly associated with histone modifications such as H3K4me3, H3K27me3, and H3K27ac. Since BRD2, BRD3, and BRD4 are essential readers of histone acetylation, blocking BET proteins disrupts histone cross-talk and downstream transcriptional activation. For example, JQ1 has been shown to abolish H3K27ac-induced H3K4me3 installation, thereby modulating gene expression [50]. Our findings suggest that BET inhibitors act as epigenetic reprogrammers, reshaping histone modifications and transcriptional networks that drive UF pathology.

**Figure 11:**
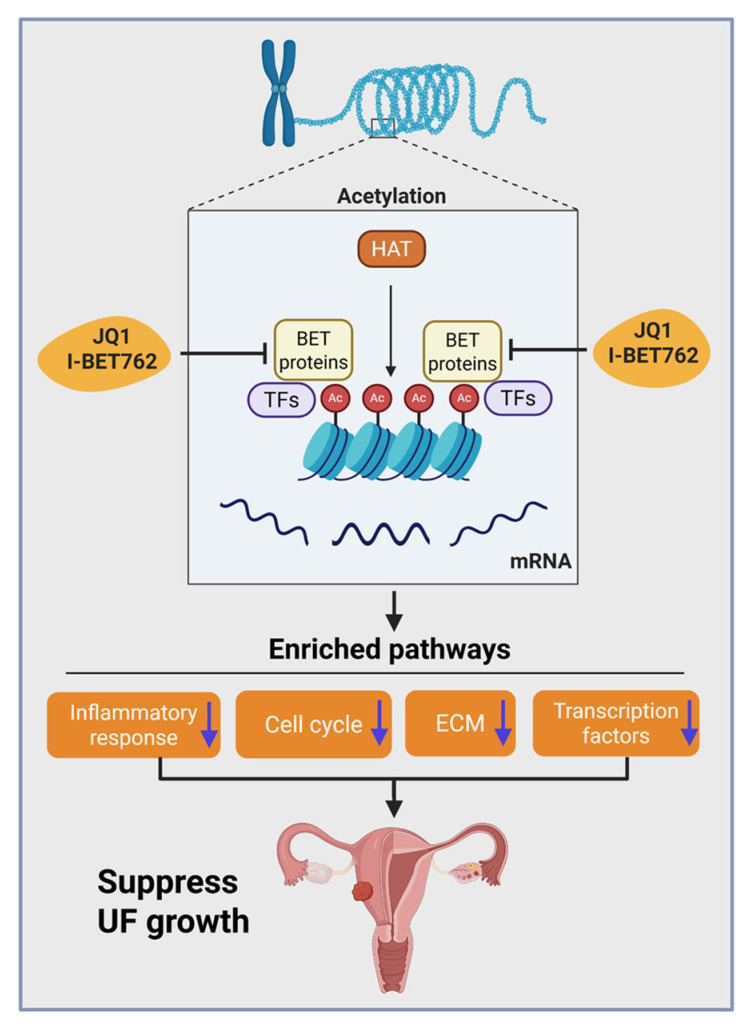
Experimental model. Our experimental model shows that targeted inhibition of BET proteins induces the enrichments of inflammatory response, cell cycle arrest, ECM deposition, and E2F targets pathways, and alters the epigenetic network in UF cells, leading to suppressing the UF growth. This figure was created using the BioRender software online app (BioRender.com). HAT: histone acetyltransferase; TFs: transcription factors; Ac: acetylation; ECM: extracellular matrix. Arrows in the boxes for inflammatory response, cell cycle, ECM, and transcription factors denote inhibition of these pathways by BETi treatment.

Based on these data, we propose a mechanistic model in which: (1) BRD2 and BRD4 are aberrantly upregulated in UFs, (2) BET inhibition suppresses UF phenotypes by reducing proliferation, inflammation, transcriptional activity, and ECM expression, (3) JQ1/I-BET762 treatment reprograms the pathological epigenome and modulates key signaling pathways, leading to inhibition of UF growth (Figure 11).

Our study has several limitations. First, the sample size is relatively small, and additional studies using a larger cohort will be necessary to more accurately determine the expression patterns of BET proteins in UF tissues. Second, the functional assays were conducted in immortalized human UF and myometrial cell lines, which do not fully recapitulate the complexity of the in vivo UF microenvironment. Therefore, further in vivo and translational studies are required to validate these findings and assess their potential clinical relevance. Third, the BET inhibitors used in this study do not delineate the specific contributions of individual BET family members to UF pathogenesis. Future experiments employing targeted knockdown or CRISPR-based approaches will be important to dissect the distinct roles of each BET protein. Lastly, although BET inhibition affected several key pathways supported by bioinformatic and RNA expression analyses, additional complementary functional assays are needed to further strengthen and confirm these observations.

In conclusion, this study provides the first evidence that BET proteins play a pivotal role in UF pathogenesis. Targeted inhibition of BET proteins suppresses UF progression by modulating cell cycle, inflammatory, transcriptional regulation, and fibrotic pathways, as well as histone modification-mediated gene regulation. Thus, BET protein inhibition represents a promising therapeutic avenue for patients with uterine fibroids.

## 4. Materials and Methods

### 4.1. Sample Collection

The study was approved by the University of Chicago’s Institutional Review Board (IRB 20–1414). Fibroid tissues were consistently collected from the peripheral parts of large intramural fibroid lesions (>5 cm in diameter) with care to avoid areas of apparent necrosis, bleeding, or degeneration. Myometrium tissues were collected at least 2 cm away from the closest fibroid lesion. Patients underwent the informed consent process, and documented informed consent forms were collected and stored. Only those records indicating that the patient had not used any hormonal treatment for at least 3 months before the surgery date were included. All tissues used in this study were collected during the secretory phase of menses. The menstrual phase designation of the endometrium was performed by a board-certified pathologist based on standard morphological and histological criteria.

### 4.2. Cells and Reagents

The immortalized human leiomyoma cell line (UtLM) and immortalized human myometrial (UtSM) cells were a generous gift from Dr. Darlene Dixon. The cells were cultured and maintained in phenol red-free, DMEM-F12 medium with 10% fetal bovine serum. In addition, BET protein inhibitors JQ1 and I-BET762 were purchased from Selleck Chemical (Cat# S7835, Houston, TX, USA) and TOCRIS (Cat# 6000, Minneapolis, MN, USA), respectively. JQ1 and I-BET762 were dissolved in DMSO to prepare 25 mM stock solutions. An equal volume of DMSO was added to the control group.

### 4.3. Protein Extraction and Immunoblot Analysis

Cells were trypsinized, washed with PBS and collected by centrifugation. The cell pellets were lysed in RIPA lysis buffer with protease and phosphatase inhibitor cocktail (Thermo Scientific, Waltham, MA, USA). The protein lysates from UF and adjacent myometrial tissues were prepared as described previously [51]. The protein was quantified using the Bradford method (Bio-Rad Protein Assay kit) (Bio-Rad Laboratories, Inc., Hercules, CA, USA). The primary antibodies, including BRD2 (5848, 1:1000 dilution, Cell Signaling Technology, Danvers, MA, USA), BRD3 (ab50818, 1:1000 dilution, Abcam, Cambridge, UK), BRD4 (83375, 1:800 dilution, Cell Signaling Technology), β-actin (A5316, 1:5000 dilution, Sigma, Saint Louis, MO, USA), Vinculin (13901, 1:2500 dilution, Cell Signaling Technology) were used. The antigen–antibody complex was detected with Trident Femto Western HRP substrate (GeneTex, Irvine, CA, USA). Specific protein bands were visualized using ChemiDoc XRS + system (Bio-Rad Laboratories Inc.). Protein band densities were quantified using the NIH ImageJ software (version 1.52r, Bethesda, MD, USA). For the quantification and statistical analysis of low-abundance BRD4 signals, the detection rate of BRD4 between groups was compared using Fisher’s exact test.

### 4.4. Cell Viability Assay

Cell viability was determined using trypan blue exclusion assay. The equal number of UF and myometrial cells (3 × 10^4^ cell/well) were seeded into 12-well tissue culture plates and treated with the BET protein inhibitors (JQ1, I-BET762) at a dose range from 1–10 µM for 48 h. This assay was performed three times in triplicate.

### 4.5. RNA Extraction and Quantitative Real-Time Polymerase Chain Reaction (qRT-PCR)

Total RNA was isolated using Trizol reagent (Invitrogen, Carlsbad, CA, USA). The concentration of total RNA was determined using NanoDrop (Thermo Scientific, Waltham, MA, USA). One microgram of total RNA from each sample was reverse transcribed to complementary DNA (cDNA) using the High-Capacity cDNA Reverse Transcription Kit (Thermo Scientific, Waltham, MA, USA).

Quantitative Real-time PCR was performed to determine the mRNA expression of genes as described previously [52]. Primers were purchased from Integrated DNA Technologies (IDT, Coralville, IA, USA) with primer sequences shown in Table 1. An equal amount of cDNA from each sample was added to the Master mix containing appropriate primer sets and SYBR green supermix (Bio-Rad Laboratories Inc.) in a 20 µL reaction volume. All samples were analyzed in triplicates. Real-time PCR analyses were performed using a Bio-Rad CFX96. Cycling conditions, including denaturation at 95 °C for 2 min. followed by 40 cycles of 95 °C for 5 s and 60 °C for 30 s then 65 °C for 5 s. Synthesis of a DNA product of the expected size was confirmed by melting curve analysis. 18S ribosomal RNA values (internal control) were used to normalize the expression data, and normalized values were used to create data graphs. Negative control was performed by running the reaction without cDNA.

### 4.6. Measurement of Cell Cycle Phase Distribution

Cell cycle phase distribution was determined by flow cytometric analysis as described previously [19]. Briefly, UF cells were cultured in DMEM/F12 medium containing 5 µM of JQ1 and I-BET762 for 48 h. Control cells were cultured in a medium containing an equal amount of DMSO. Cells were then washed with PBS, fixed in 70% ethanol, and hypotonically lysed in 1 mL of DNA staining solution [0.05 mg/mL PI (Sigma) and 0.1%Triton X-100]. The cell cycle data were analyzed with an Epics XL-MCL flow cytometer (Beckman Coulter, Miami, FL, USA), with System II (version 3.0) software (Beckman Coulter, Indianapolis, IN, USA).

### 4.7. RNA-Sequencing

To determine the mechanism underlying the inhibitory effect of BET inhibition on the UFs, RNA-seq was performed. Briefly, the UtLM cells were treated with BET inhibitor JQ1 (5 µM, n = 3), I-BET762 (5 µM, n = 4), and DMSO vehicle control (n = 4) for 48 h. RNA was isolated using Trizol and treated with DNaseI. RNA quality and quantity were assessed using the Agilent Bioanalyzer. Strand-specific RNA-SEQ libraries were prepared using a TruSEQ total RNA-SEQ library protocol (Illumina provided). Library quality and quantity were assessed using the Agilent Bioanalyzer (Santa Clara, CA, USA), and libraries were sequenced using an Illumina NovaSEQ6000 (San Diego, CA, USA).

### 4.8. Transcriptome Profiles Analysis

#### 4.8.1. Transcriptome Data Analysis

Transcriptome data analysis was carried out using a variety of R packages. The quality of reads was controlled using FastQC (http://www.bioinformatics.babraham.ac.uk/projects/fastqc (accessed on 11 February 2023)), version 0.73, and then the reads were processed using Trimmomatic [53]. The version and parameters were chosen to trim the reads according to the FastQC results. The reads were mapped to the human reference genome, version hg38, using Hisat2 [54], version 2.2.1. Next, raw reads were mapped to the human reference transcriptome using FeatureCounts [55], version 2.0.3, and the annotation file of Gencode [56], version V41. Gene counts were pre-processed and normalized using the DESeq2 [57] package in R, version 1.36.0. The quality of samples was examined using a comparative boxplot and PCA, and outlier samples were excluded from the analysis. DEGs were identified using DESeq2.

#### 4.8.2. Functional Enrichment Analysis

Gene set enrichment analysis (GSEA) preranked [58] was performed using the fgsea R package, version 1.22.0, with gene set collections downloaded from the Molecular Signatures Database (MSigDB v7.5.1 for H (hallmark gene sets) and C2 (curated gene sets)). The significant pathways were determined based on the parameters, including n = 1000 permutations, where *p*-adjust < 0.05, and FDR < 0.05. The Enrichplot (https://yulab-smu.top/biomedical-knowledge-mining-book (accessed on 21 May 2023)) R package, version 1.16.2, was utilized to visualize the results. Additionally, we conducted histone modification enrichment analysis using ENCODE Histone Modifications 2015 in EnrichR [59] through clusterProfiler [60], version 4.4.4, to uncover the epigenetic mechanisms underlying the regulation of DEGs.

### 4.9. Statistical Analysis

All experiments were conducted with at least three biological replicates. A comparison of two groups was carried out using a Student *t*-test for parametric distribution and a Mann–Whitney test for nonparametric distribution. Comparison of multiple groups was carried out by analysis of variance (ANOVA) followed by a post-test using Tukey’s for parametric distribution and Kruskal–Wallis test followed by a Dunn’s post-test for nonparametric distribution, using GraphPad Prism 5 software. Data was presented as mean ± standard error of the mean (SEM).

## Figures and Tables

**Table 1:** Primers used in this study.

Gene Symbol	Sequence	F or R	Assay	Species	Amplicon Size (bp)	Accession
*BRD2*	GCCCATGAGTTACGATGAGAAG	F	q-PCR	Human	101	NM_005104.4
*BRD2*	GCTCCCTGGCTTGGATTATATG	R	q-PCR	Human		
*BRD3*	AACCACTTCCCGAGCTTATG	F	q-PCR	Human	118	NM_007371.4
*BRD3*	TCTCTGCGACTGTGTGAATG	R	q-PCR	Human		
*BRD4*	GAAGACTCCGAAACAGAGATGG	F	q-PCR	Human	93	AF386649.1
*BRD4*	CTGCTGATGGTGGTGATGAT	R	q-PCR	Human		
*PCNA*	GGACACTGCTGGTGGTATTT	F	q-PCR	Human	105	J04718
*PCNA*	CAGAACTGGTGGAGGGTAAAC	R	q-PCR	Human		
*CCND1*	GGGTTGTGCTACAGATGATAGAG	F	q-PCR	Human	112	NM-053056.3
*CCND1*	AGACGCCTCCTTTGTGTTAAT	R	q-PCR	Human		
*CDK1*	TCAGTCTTCAGGATGTGCTTATG	F	q-PCR	Human	107	NM_001786.5
*CDK1*	GTACTGACCAGGAGGGATAGAA	R	q-PCR	Human		
*STING1*	GGTGCCTGATAACCTGAGTATG	F	q-PCR	Human	103	NM_198282.4
*STING1*	GCTGTAAACCCGATCCTTGA	R	q-PCR	Human		
*SIRT1*	AGAACCCATGGAGGATGAAAG	F	q-PCR	Human	111	AF083106.2
*SIRT1*	TCATCTCCATCAGTCCCAAATC	R	q-PCR	Human		
*DNMT3A*	CTGAGGTAGCGACACAAAGTTA	F	q-PCR	Human	101	NM_175629.2
*DNMT3A*	CTCTTCTGGGTGCTGATACTTC	R	q-PCR	Human		
*DNMT1*	CGGCCTCATCGAGAAGAATATC	F	q-PCR	Human	95	NM_001130823.3
*DNMT1*	TGCCATTAACACCACCTTCA	R	q-PCR	Human		
*NF-kB*	GTGACAGGAGACGTGAAGATG	F	q-PCR	Human	104	NM_003998.4
*NF-kB*	TGAAGGTGGATGATTGCTAAGT	R	q-PCR	Human		
*18S*	CACGGACAGGATTGACAGATT	F	q-PCR	Human	119	NR_145820
*18S*	GCCAGAGTCTCGTTCGTTATC	R	q-PCR	Human		

## Data Availability

Raw FASTQ files were deposited in the NCBI Gene Expression Omnibus (GSE281309).

## Supplementary Materials

The following supporting information can be downloaded at: https://www.mdpi.com/article/10.3390/ijms262412144/s1.

## Abbreviations

The following abbreviations are used in this manuscript:

Ac	Acetylation
BET	Bromodomain and Extra-Terminal domain
BETi	BET inhibitor
BCL2	B-cell lymphoma 2
BRD	Bromodomain-containing proteins 2, 3, and 4
CCND1	Cyclin D1
CDK	Cyclin-dependent kinase
CDKN1A	Cyclin-dependent kinase inhibitor 1A (p21)
CDKN1B	Cyclin-dependent kinase inhibitor 1B (p27)
CGAS	Cyclic GMP–AMP synthase
COL	Collagen
CPM	Counts Per Million
CXCL	C-X-C motif chemokine ligand
DEGs	Differentially expressed genes
DNMT	DNA methyltransferases
ECM	Extracellular matrix
E2F	E2F transcription factor
EZH2	Enhancer of zeste homolog 2
FDR	False discovery rate
HAT	Histone acetyltransferase
UtLM	Immortalized uterine fibroid (leiomyoma) cell line
I-BET762	A selective BET inhibitor
ImageJ	Image processing and quantification software
JQ1	A small molecule BET inhibitor
LOXL	Lysyl oxidase–like proteins
MKI67	Gene encoding the Ki-67 proliferation marker
MSigDB	Molecular Signatures Database
NF-κB1	Nuclear factor kappa-B subunit 1
PCNA	Proliferating cell nuclear antigen
qPCR	Quantitative polymerase chain reaction
RNA-seq	RNA sequencing
SEM	Standard error of the mean
STING1	Stimulator of interferon genes 1
TGFβ3	Transforming growth factor beta 3
UF	Uterine fibroid
UtSM	Immortalized uterine myometrial cell line

## References

[1] Yang Q., Ciebiera M., Victoria Bariani M., Ali M., Elkafas H., Boyer T.G., Al-Hendy A. (2021). Comprehensive Review of Uterine Fibroids: Developmental Origin, Pathogenesis, and Treatment. Endocr. Rev..

[2] Bulun S.E. (2013). Uterine fibroids. N. Engl. J. Med..

[3] Stewart E.A., Laughlin-Tommaso S.K., Catherino W.H., Lalitkumar S., Gupta D., Vollenhoven B. (2016). Uterine fibroids. Nat. Rev. Dis. Primers.

[4] Whynott R.M., Vaught K.C.C., Segars J.H. (2017). The Effect of Uterine Fibroids on Infertility: A Systematic Review. Semin. Reprod. Med..

[5] Hazimeh D., Coco A., Casubhoy I., Segars J., Singh B. (2024). The Annual Economic Burden of Uterine Fibroids in the United States (2010 Versus 2022): A Comparative Cost-Analysis. Reprod. Sci..

[6] Yang Q., Mas A., Diamond M.P., Al-Hendy A. (2016). The Mechanism and Function of Epigenetics in Uterine Leiomyoma Development. Reprod. Sci..

[7] Berta D.G., Kuisma H., Valimaki N., Raisanen M., Jantti M., Pasanen A., Karhu A., Kaukomaa J., Taira A., Cajuso T. (2021). Deficient H2A.Z deposition is associated with genesis of uterine leiomyoma. Nature.

[8] Jain A.K., Barton M.C. (2017). Bromodomain Histone Readers and Cancer. J. Mol. Biol..

[9] Winter G.E., Mayer A., Buckley D.L., Erb M.A., Roderick J.E., Vittori S., Reyes J.M., di Iulio J., Souza A., Ott C.J. (2017). BET Bromodomain Proteins Function as Master Transcription Elongation Factors Independent of CDK9 Recruitment. Mol. Cell.

[10] Bai L., Zhou B., Yang C.Y., Ji J., McEachern D., Przybranowski S., Jiang H., Hu J., Xu F., Zhao Y. (2017). Targeted Degradation of BET Proteins in Triple-Negative Breast Cancer. Cancer Res..

[11] Picaud S., Leonards K., Lambert J.P., Dovey O., Wells C., Fedorov O., Monteiro O., Fujisawa T., Wang C.Y., Lingard H. (2016). Promiscuous targeting of bromodomains by bromosporine identifies BET proteins as master regulators of primary transcription response in leukemia. Sci. Adv..

[12] Shigeta S., Lui G.Y.L., Shaw R., Moser R., Gurley K.E., Durenberger G., Rosati R., Diaz R.L., Ince T.A., Swisher E.M. (2021). Targeting BET Proteins BRD2 and BRD3 in Combination with PI3K-AKT Inhibition as a Therapeutic Strategy for Ovarian Clear Cell Carcinoma. Mol. Cancer Ther..

[13] Mazur P.K., Herner A., Mello S.S., Wirth M., Hausmann S., Sanchez-Rivera F.J., Lofgren S.M., Kuschma T., Hahn S.A., Vangala D. (2015). Combined inhibition of BET family proteins and histone deacetylases as a potential epigenetics-based therapy for pancreatic ductal adenocarcinoma. Nat. Med..

[14] Faivre E.J., McDaniel K.F., Albert D.H., Mantena S.R., Plotnik J.P., Wilcox D., Zhang L., Bui M.H., Sheppard G.S., Wang L. (2020). Selective inhibition of the BD2 bromodomain of BET proteins in prostate cancer. Nature.

[15] Gilan O., Rioja I., Knezevic K., Bell M.J., Yeung M.M., Harker N.R., Lam E.Y.N., Chung C.W., Bamborough P., Petretich M. (2020). Selective targeting of BD1 and BD2 of the BET proteins in cancer and immunoinflammation. Science.

[16] Tiago M., Capparelli C., Erkes D.A., Purwin T.J., Heilman S.A., Berger A.C., Davies M.A., Aplin A.E. (2020). Targeting BRD/BET proteins inhibits adaptive kinome upregulation and enhances the effects of BRAF/MEK inhibitors in melanoma. Br. J. Cancer.

[17] Ozer H.G., El-Gamal D., Powell B., Hing Z.A., Blachly J.S., Harrington B., Mitchell S., Grieselhuber N.R., Williams K., Lai T.H. (2018). BRD4 Profiling Identifies Critical Chronic Lymphocytic Leukemia Oncogenic Circuits and Reveals Sensitivity to PLX51107, a Novel Structurally Distinct BET Inhibitor. Cancer Discov..

[18] Leal A.S., Williams C.R., Royce D.B., Pioli P.A., Sporn M.B., Liby K.T. (2017). Bromodomain inhibitors, JQ1 and I-BET 762, as potential therapies for pancreatic cancer. Cancer Lett..

[19] Jiang G., Deng W., Liu Y., Wang C. (2020). General mechanism of JQ1 in inhibiting various types of cancer. Mol. Med. Rep..

[20] Islam M.S., Ciavattini A., Petraglia F., Castellucci M., Ciarmela P. (2018). Extracellular matrix in uterine leiomyoma pathogenesis: A potential target for future therapeutics. Hum. Reprod. Update.

[21] Islam M.S., Afrin S., Singh B., Jayes F.L., Brennan J.T., Borahay M.A., Leppert P.C., Segars J.H. (2021). Extracellular matrix and Hippo signaling as therapeutic targets of antifibrotic compounds for uterine fibroids. Clin. Transl. Med..

[22] Jamaluddin M.F.B., Nahar P., Tanwar P.S. (2018). Proteomic Characterization of the Extracellular Matrix of Human Uterine Fibroids. Endocrinology.

[23] Yang Q., Al-Hendy A. (2023). Update on the Role and Regulatory Mechanism of Extracellular Matrix in the Pathogenesis of Uterine Fibroids. Int. J. Mol. Sci..

[24] Leppert P.C., Jayes F.L., Segars J.H. (2014). The extracellular matrix contributes to mechanotransduction in uterine fibroids. Obstet. Gynecol. Int..

[25] Yang Q., Falahati A., Khosh A., Vafaei S., Al-Hendy A. (2024). Targeting Bromodomain-Containing Protein 9 in Human Uterine Fibroid Cells. Reprod. Sci..

[26] Yang Q., Vafaei S., Falahati A., Khosh A., Bariani M.V., Omran M.M., Bai T., Siblini H., Ali M., He C. (2024). Bromodomain-Containing Protein 9 Regulates Signaling Pathways and Reprograms the Epigenome in Immortalized Human Uterine Fibroid Cells. Int. J. Mol. Sci..

[27] Bert S.A., Robinson M.D., Strbenac D., Statham A.L., Song J.Z., Hulf T., Sutherland R.L., Coolen M.W., Stirzaker C., Clark S.J. (2013). Regional activation of the cancer genome by long-range epigenetic remodeling. Cancer Cell..

[28] Schrump D.S., Hong J.A., Nguyen D.M. (2007). Utilization of chromatin remodeling agents for lung cancer therapy. Cancer J..

[29] Qi J. (2014). Bromodomain and extraterminal domain inhibitors (BETi) for cancer therapy: Chemical modulation of chromatin structure. Cold Spring Harb. Perspect. Biol..

[30] Kaur J., Daoud A., Eblen S.T. (2019). Targeting Chromatin Remodeling for Cancer Therapy. Curr. Mol. Pharmacol..

[31] Magnani L., Stoeck A., Zhang X., Lanczky A., Mirabella A.C., Wang T.L., Gyorffy B., Lupien M. (2013). Genome-wide reprogramming of the chromatin landscape underlies endocrine therapy resistance in breast cancer. Proc. Natl. Acad. Sci. USA.

[32] Magic Z., Supic G., Brankovic-Magic M. (2009). Towards targeted epigenetic therapy of cancer. J. BUON.

[33] Zhao R., Casson A.G. (2008). Epigenetic aberrations and targeted epigenetic therapy of esophageal cancer. Curr. Cancer Drug Targets.

[34] Bai H., Cao D., Yang J., Li M., Zhang Z., Shen K. (2016). Genetic and epigenetic heterogeneity of epithelial ovarian cancer and the clinical implications for molecular targeted therapy. J. Cell Mol. Med..

[35] Shimamura T., Chen Z., Soucheray M., Carretero J., Kikuchi E., Tchaicha J.H., Gao Y., Cheng K.A., Cohoon T.J., Qi J. (2013). Efficacy of BET bromodomain inhibition in Kras-mutant non-small cell lung cancer. Clin. Cancer Res..

[36] Kim Y.H., Kim M., Kim J.E., Yoo M., Lee H.K., Lee C.O., Yoo M., Jung K.Y., Kim Y., Choi S.U. (2021). Novel brd4 inhibitors with a unique scaffold exhibit antitumor effects. Oncol. Lett..

[37] Funck-Brentano E., Vizlin-Hodzic D., Nilsson J.A., Nilsson L.M. (2021). BET bromodomain inhibitor HMBA synergizes with MEK inhibition in treatment of malignant glioma. Epigenetics.

[38] Sanders Y.Y., Lyv X., Zhou Q.J., Xiang Z., Stanford D., Bodduluri S., Rowe S.M., Thannickal V.J. (2020). Brd4-p300 inhibition downregulates Nox4 and accelerates lung fibrosis resolution in aged mice. JCI Insight.

[39] Chakraborty D., Benham V., Jdanov V., Bullard B., Leal A.S., Liby K.T., Bernard J.J. (2018). A BET Bromodomain Inhibitor Suppresses Adiposity-Associated Malignant Transformation. Cancer Prev. Res..

[40] Qiu H., Jackson A.L., Kilgore J.E., Zhong Y., Chan L.L., Gehrig P.A., Zhou C., Bae-Jump V.L. (2015). JQ1 suppresses tumor growth through downregulating LDHA in ovarian cancer. Oncotarget.

[41] Kamalipooya S., Zarezadeh R., Latifi Z., Nouri M., Fattahi A., Salemi Z. (2021). Serum transforming growth factor beta and leucine-rich alpha-2-glycoprotein 1 as potential biomarkers for diagnosis of uterine leiomyomas. J. Gynecol. Obstet. Hum. Reprod..

[42] Ciebiera M., Wlodarczyk M., Wrzosek M., Meczekalski B., Nowicka G., Lukaszuk K., Ciebiera M., Slabuszewska-Jozwiak A., Jakiel G. (2017). Role of Transforming Growth Factor beta in Uterine Fibroid Biology. Int. J. Mol. Sci..

[43] Lewis T.D., Malik M., Britten J., Parikh T., Cox J., Catherino W.H. (2019). Ulipristal acetate decreases active TGF-beta3 and its canonical signaling in uterine leiomyoma via two novel mechanisms. Fertil. Steril..

[44] Norian J.M., Malik M., Parker C.Y., Joseph D., Leppert P.C., Segars J.H., Catherino W.H. (2009). Transforming growth factor beta3 regulates the versican variants in the extracellular matrix-rich uterine leiomyomas. Reprod. Sci..

[45] Belkina A.C., Nikolajczyk B.S., Denis G.V. (2013). BET protein function is required for inflammation: Brd2 genetic disruption and BET inhibitor JQ1 impair mouse macrophage inflammatory responses. J. Immunol..

[46] Kondo Y. (2009). Epigenetic cross-talk between DNA methylation and histone modifications in human cancers. Yonsei Med. J..

[47] Winter S., Fischle W. (2010). Epigenetic markers and their cross-talk. Essays Biochem..

[48] Szulwach K.E., Li X., Smrt R.D., Li Y., Luo Y., Lin L., Santistevan N.J., Li W., Zhao X., Jin P. (2010). Cross talk between microRNA and epigenetic regulation in adult neurogenesis. J. Cell Biol..

[49] Lopez G., Song Y., Lam R., Ruder D., Creighton C.J., Bid H.K., Bill K.L., Bolshakov S., Zhang X., Lev D. (2016). HDAC Inhibition for the Treatment of Epithelioid Sarcoma: Novel Cross Talk Between Epigenetic Components. Mol. Cancer Res..

[50] Zhao W., Xu Y., Wang Y., Gao D., King J., Xu Y., Liang F.S. (2021). Investigating crosstalk between H3K27 acetylation and H3K4 trimethylation in CRISPR/dCas-based epigenome editing and gene activation. Sci. Rep..

[51] Yang Q., Nair S., Laknaur A., Ismail N., Diamond M.P., Al-Hendy A. (2016). The Polycomb Group Protein EZH2 Impairs DNA Damage Repair Gene Expression in Human Uterine Fibroids. Biol. Reprod..

[52] Yang Q., Elam L., Laknaur A., Gavrilova-Jordan L., Lue J., Diamond M.P., Al-Hendy A. (2015). Altered DNA repair genes in human uterine fibroids are epigenetically regulated via EZH2 histone methyltransferase. Fertil. Steril..

[53] Bolger A.M., Lohse M., Usadel B. (2014). Trimmomatic: A flexible trimmer for Illumina sequence data. Bioinformatics.

[54] Kim D., Paggi J.M., Park C., Bennett C., Salzberg S.L. (2019). Graph-based genome alignment and genotyping with HISAT2 and HISAT-genotype. Nat. Biotechnol..

[55] Liao Y., Smyth G.K., Shi W. (2014). featureCounts: An efficient general purpose program for assigning sequence reads to genomic features. Bioinformatics.

[56] Harrow J., Frankish A., Gonzalez J.M., Tapanari E., Diekhans M., Kokocinski F., Aken B.L., Barrell D., Zadissa A., Searle S. (2012). GENCODE: The reference human genome annotation for The ENCODE Project. Genome Res..

[57] Love M.I., Huber W., Anders S. (2014). Moderated estimation of fold change and dispersion for RNA-seq data with DESeq2. Genome Biol..

[58] Subramanian A., Tamayo P., Mootha V.K., Mukherjee S., Ebert B.L., Gillette M.A., Paulovich A., Pomeroy S.L., Golub T.R., Lander E.S. (2005). Gene set enrichment analysis: A knowledge-based approach for interpreting genome-wide expression profiles. Proc. Natl. Acad. Sci. USA.

[59] Kuleshov M.V., Jones M.R., Rouillard A.D., Fernandez N.F., Duan Q., Wang Z., Koplev S., Jenkins S.L., Jagodnik K.M., Lachmann A. (2016). Enrichr: A comprehensive gene set enrichment analysis web server 2016 update. Nucleic Acids Res..

[60] Yu G., Wang L.G., Han Y., He Q.Y. (2012). clusterProfiler: An R package for comparing biological themes among gene clusters. OMICS.

