# Supplementary material for: Decoding Bromodomain and Extra-Terminal Domain Protein-Mediated Epigenetic Mechanisms in Human Uterine Fibroids
Source: Int J Mol Sci. 2025 Dec 17;26(24):12144. doi: 10.3390/ijms262412144 (PMC12733843; doi:10.3390/ijms262412144)
Supplement: Supplementary file 1 [file ijms-26-12144-s001.zip › ijms-4008956-supplementary.pdf]

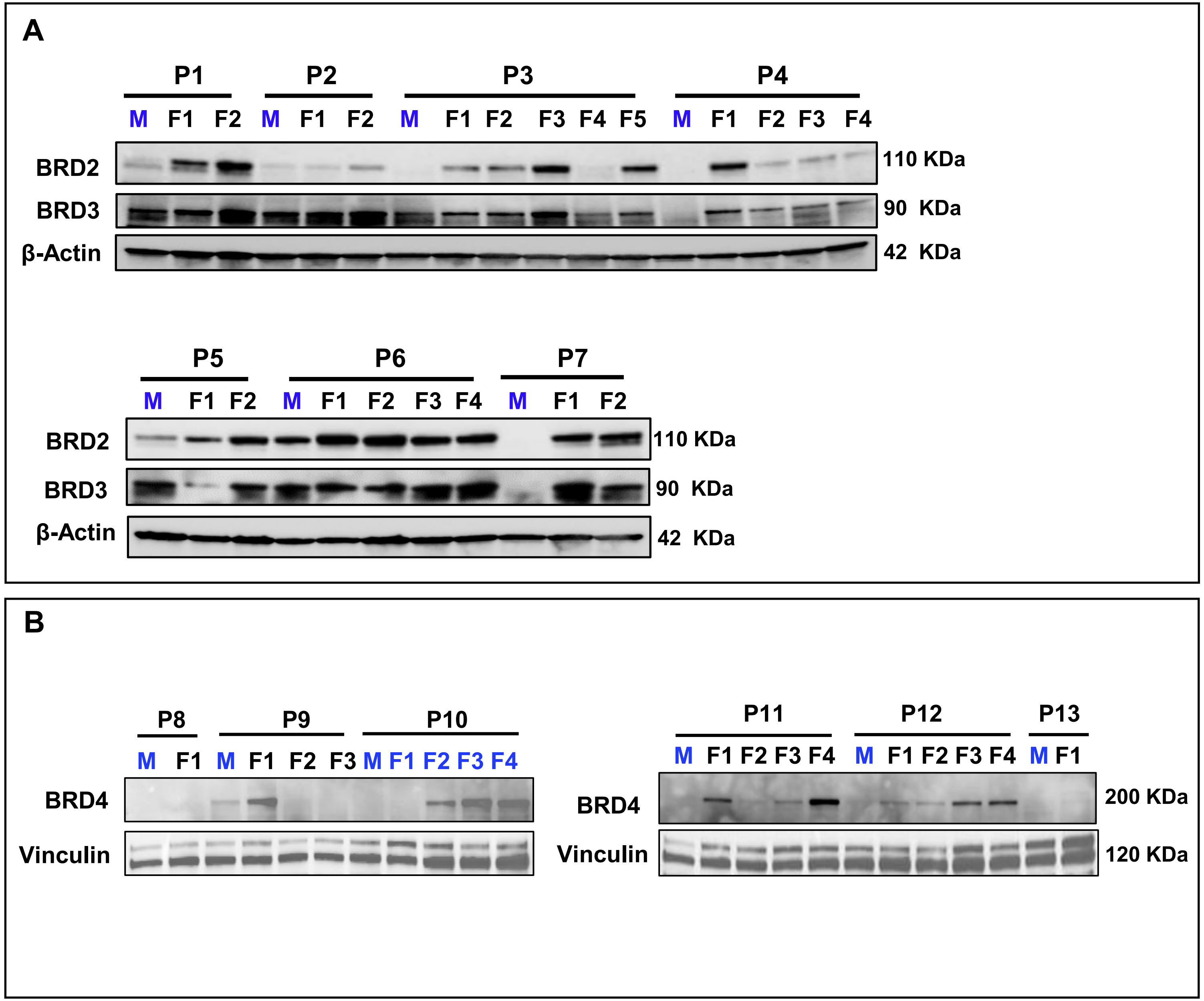

Supplemental Figure S1

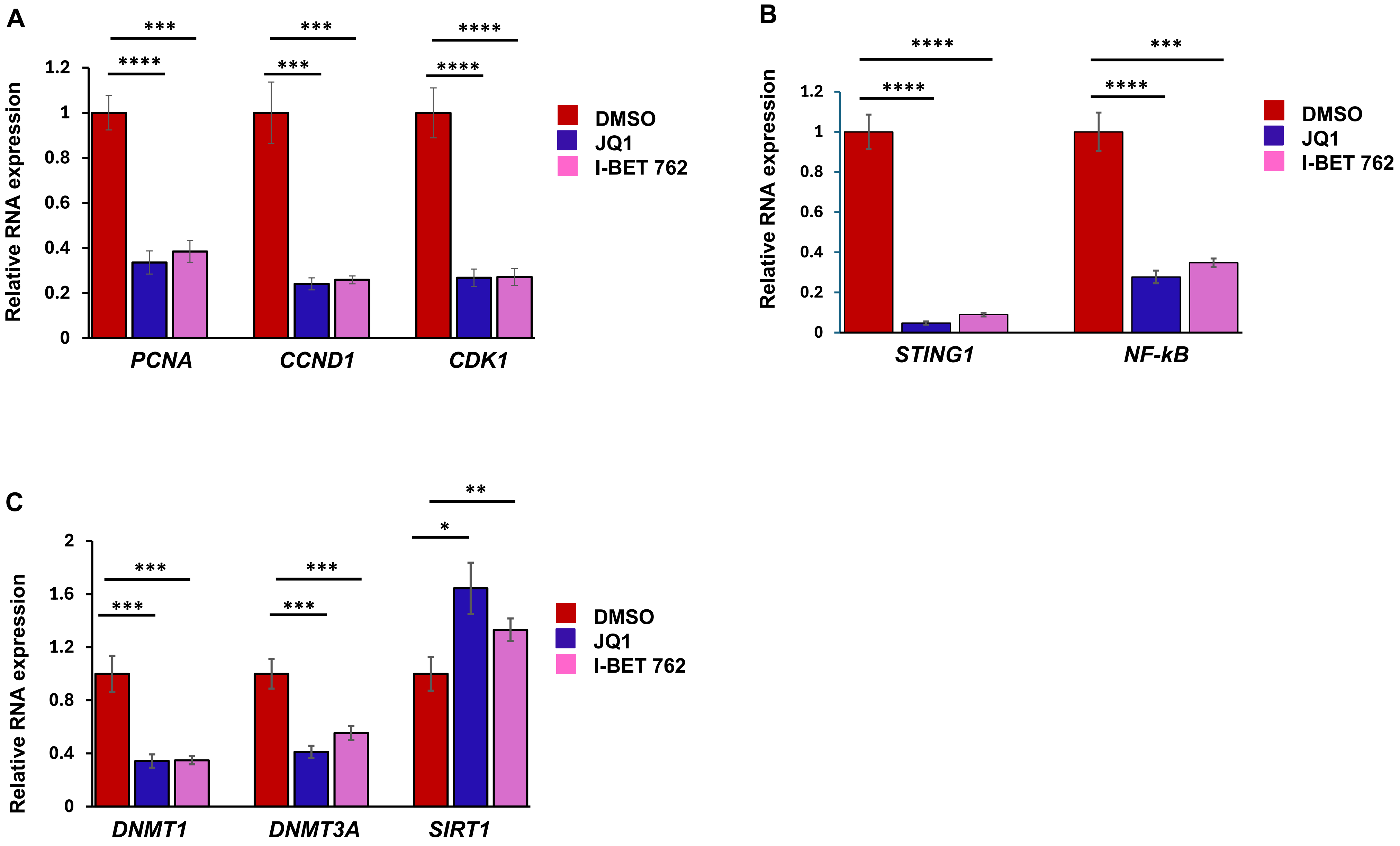

Supplemental Figure S2

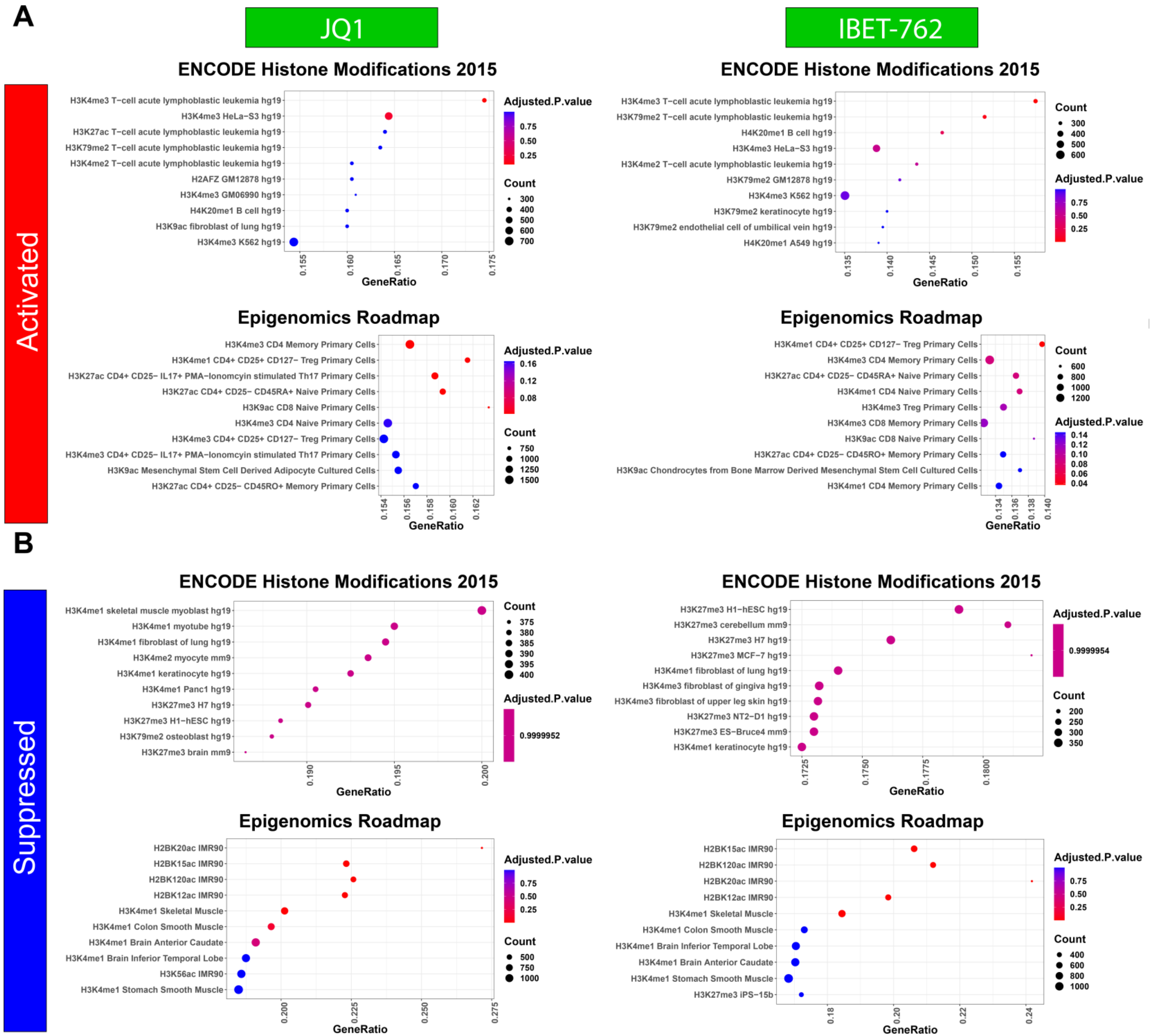

Supplemental Figure S3
